# Small Molecule Compounds Identified from Mixture-Based Library Inhibit Binding between *Plasmodium falciparum* Infected Erythrocytes and Endothelial Receptor ICAM-1

**DOI:** 10.3390/ijms22115659

**Published:** 2021-05-26

**Authors:** Olga Chesnokov, Pimnitah Visitdesotrakul, Komal Kalani, Adel Nefzi, Andrew V. Oleinikov

**Affiliations:** 1 Charles E. Schmidt College of Medicine, Florida Atlantic University, Boca Raton, FL 33428, USA; onchesnokova@yahoo.com (O.C.); pvisitde@fau.edu (P.V.); 2 Center for Translational Science, Florida International University (FIU), Port Saint Lucie, FL 34987, USA; kkalani@fiu.edu

**Keywords:** *P. falciparum*, severe and cerebral malaria, small molecule inhibitors, combinatorial chemistry, mixture-based libraries, drug discovery, solid-phase synthesis, thiazoles

## Abstract

Specific adhesion of *P. falciparum* parasite-infected erythrocytes (IE) in deep vascular beds can result in severe complications, such as cerebral malaria, placental malaria, respiratory distress, and severe anemia. Cerebral malaria and severe malaria syndromes were associated previously with sequestration of IE to a microvasculature receptor ICAM-1. The screening of Torrey Pines Scaffold Ranking library, which consists of more than 30 million compounds designed around 75 molecular scaffolds, identified small molecules that inhibit cytoadhesion of ICAM-1-binding IE to surface-immobilized receptor at IC_50_ range down to ~350 nM. With their low cytotoxicity toward erythrocytes and human endothelial cells, these molecules might be suitable for development into potentially effective adjunct anti-adhesion drugs to treat cerebral and/or severe malaria syndromes. Our two-step high-throughput screening approach is specifically designed to work with compound mixtures to make screening and deconvolution to single active compounds fast and efficient.

## 1. Introduction

Malaria caused by *Plasmodium falciparum* parasite is one of the major deadly diseases in the world, with a yearly count of half a billion cases and 0.4 million deaths, mostly among young children [1]. Most deaths result from severe malaria (SM) complications that include cerebral malaria (CM), placental malaria (PM), respiratory distress, and severe anemia. The spread of parasites resistant to the artemisinin family of compounds [2] threatens recent progress achieved by antimalarial campaigns and underscores the urgent need to identify new anti-malarial drugs. Even more important, though efficient anti-malarial drugs rapidly kill parasites, significant mortality (10–15%) still results from SM even in hospital admissions, particularly within 24 h, likely because continued cytoadherence of infected erythrocytes (IE) long after the parasite has been killed [3]. Unfortunately, novel parasite-killing drugs will have the same problem if they do not prevent or reverse IE sequestration. Cytoadherence [4,5,6] plays an important role in the lifecycle and virulence of *P. falciparum*, the deadliest of human malaria species, and is a cause of at least placental malaria (PM) [7] and cerebral malaria (CM) [8,9,10,11,12].

The role of sequestration of IE to intercellular adhesion molecule 1 (ICAM-1) receptor in CM was suggested some time ago based on its overexpression and co-localization with sequestered IE in the brain endothelium of CM patients [10]. Members of a large protein family, *Plasmodium falciparum* Erythrocyte Membrane Protein 1 (PfEMP1s), which express one at a time on the surface of IE [13,14], were implicated as major molecules that are involved in IE sequestration (reviewed in Reference [15]). Only six DBLβ domains from ~60 PfEMP1 proteins containing more than 250 domains in the *P. falciparum* IT4 strain have been identified as binding to ICAM1 [16] and only two DBLβ domains in the 3D7 strain [17,18]. The fact that only a small number of PfEMP1 domains strongly bind ICAM-1 fits well with the epidemiology of CM, which is a rare but deadly severe malaria syndrome. Extensive epidemiology data [19,20] support the hypothesis that only a limited number of parasite lines, possibly expressing particular variants of PfEMP1 (containing particular domains), may be the source of severe disease, in a similar fashion to PM. Thus, specific DBLβ domains that bind to ICAM-1 [16,17,18,21] with high avidity in low nanomolar range [12,22] are perfect candidates for the role in CM and, more broadly, in SM [23,24]. In addition, it has been suggested recently, that predominantly those PfEMP1 proteins that contain tandem of domains biding to endothelial protein C receptor (EPCR) and ICAM-1 receptor are involved in CM pathogenesis [25,26], potentially binding through one or both receptors [12,27], though the role of EPCR in this binding is still controversial [28]. In addition, the expression level of EPCR in the brain microvessel endothelium—the place of IE sequestration in CM—is poor [29]. Even if both receptors are involved in CM pathological process, this may indicate a major role for ICAM-1 receptor, at least in initial strong binding to brain microvascular endothelial cells; however, parasites expressing PfEMP-1s with EPCR-binding domains (within DC8 cassette) non-linked to ICAM-1-binding domains have been associated with pediatric CM when evaluated by retinopathy or brain-swelling status [30]. 

It would be logical to suggest that negating cytoadherence would have a strong positive effect on the host’s fight *P. falciparum* against infection, thus justifying the need for adjunctive therapies to prevent and/or reverse the pathophysiological consequences of cytoadherence in SM cases. In addition, these drugs with broad anti-adhesion activity might be potentially useful as adjunct treatment during anti-malarial vaccine immunizations in endemic areas by preventing/reducing IE sequestration in infected individuals and, thus, providing better exposure of IE to immune system. Herein, we report the identification of novel small-molecule inhibitors of cytoadhesion of *P. falciparum* parasite-infected erythrocytes to ICAM-1, the host receptor involved in CM, from Torrey Pines at Florida International University (TP-FIU) Mixture-Based Combinatorial Chemical Libraries.

Earlier, we developed a high-throughput approach to identify anti-adhesion compounds that inhibit binding of IE to endothelial receptors [31]. Using this approach, we identified three compounds from screening 10,000 Chembridge small-molecule compound library that inhibit in vitro cytoadhesion of ICAM-1-binding *P. falciparum* parasite line 3G8 to surface-immobilized ICAM-1 receptor [31]. As the screened library was small in size, which significantly limits the number of active hits, we identified compounds with a modest IC_50_ values in micromolar range. To find more potent cytoadhesion inhibitors, in this work, we employed TPI libraries containing 30 million compounds [32] to identify two compounds with IC_50_ values in nM range. These identified novel active small-molecule compounds that inhibit heterologous *P. falciparum* IE line binding to ICAM-1 are strong candidates for development into anti-cytoadhesion drugs for treatment of SM and/or CM.

## 2. Results 

### 2.1. Screening of the Mixtures Library and Deconvolution of Hit Mixtures to Individual Compounds

**Figure 1:**
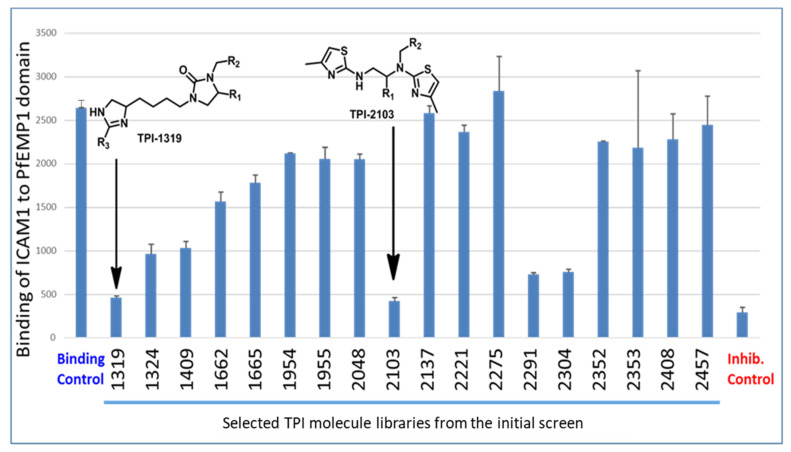
Confirmation of hits from initial screen using selected inhibiting and non-inhibiting libraries. Graph shows binding of ICAM-1receptor in solution to a bead-bound PfEMP1 PF11_0521 DBL2β3 domain. Binding control—no compound library added, binding buffer contains the same amount of DMF as samples with compound libraries. Inhibition control—ICAM-1 receptor pre-incubated with mAb My-13 [31]. Dilution of each library is 1:10. AFU, Arbitrary fluorescence units. Error bars are SD from duplicates. All inhibitions are highly significant (one-way ANOVA, Dunnett’s multiple comparison test, *p* < 0.0001 for TPI 1319, 1324, 1409, 2103, 2291, 2304, and inhibition control; *p* < 0.001 for TPI 1662, *p* < 0.01 for TPI 1665).

We screened the TP Scaffold Ranking library plate, which consists of more than 30 million compounds designed around 75 molecular scaffolds systematically arranged in positional scanning and scaffold ranking formats. Each mixture sample contains an equal molar concentration of every compound of a given core scaffold (total concentration 250 μg/mL). We conducted the screening of the scaffold plate at two concentrations (dilution 1:100 and 1:10), using our previously described procedure [31] and PF11_0521 DBL2β3 domain from 3D7 line PfEMP1 protein [17,22,31] for ICAM-1 binding inhibition. Moreover, to select for compounds that specifically bind to PfEMP1 domain and not to receptor to exert their binding-inhibitory activity, we washed drugs off after their incubation with the domain and then added ICAM-1 receptor for interaction. This is important, as it allows for the identification of compounds inhibiting interactions between ICAM-1 receptor and IEs but not between ICAM-1 and its natural ligands, to reduce potential side effects. This also allows for the selection of stronger binding compounds and reduces their number. While little inhibition was observed at 1:100 dilution, several libraries were strongly inhibiting at 1:10 dilution (total concentration, ~50 μM; concentration of individual compounds in each assay, ~10 nM to 10 pM, depending on the mixture). These hit libraries (and a few non-inhibiting) were retested and confirmed the initial results (Figure 1).

**Figure 2:**
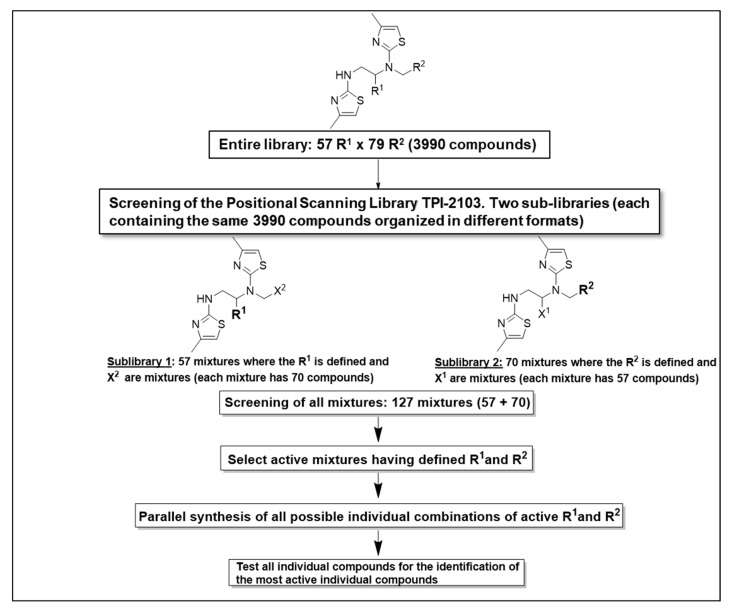
Deconvolution of the positional scan dithiazole library TPI-2103 and synthesis of individual compounds for the identification of the most active hits.

Based on these results (Figure 1, scaffold TPI-2103), the dithiazole Positional Scanning Synthetic Combinatorial Library shown in Figure 2 (TPI-2103, 3990 compounds) was selected for positional scanning deconvolution of binding inhibiting compounds. As outlined in Figure 2 and Supplementary Figure S1, this library, having two sites of diversity, consists of two separate sub-libraries, each having a single defined position (R) and mixtures positions (X). The figure also illustrates the approach to identify the most active groups at each position of this library directly from the screening data. The screening of the two sets of sub-libraries (70 + 57) provided information about the most active groups of each variable position in the TPI-2103 library.

**Figure 3:**
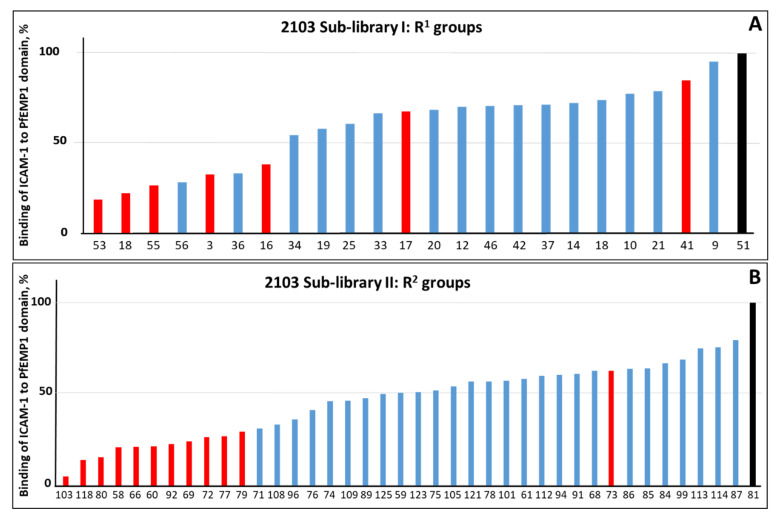
Inhibition of ICAM-1 binding to bead-immobilized PF11_0521 DBL2β3 domain by twenty-four mixtures (out of 50) with R^1^ (**A**) and forty mixtures (out of 70) with R^2^ (**B**) groups in TPI-2103 with highest binding inhibiting activity. Mixtures were assayed at concentration 2.5 μg/mL. We measured 100% binding in the absence of compounds, using binding buffer supplemented with the same concentration of DMF as samples with compounds. All groups selected for the synthesis of single compounds library (TPI-2648) are indicated by red color (active) and by black color (inactive). List of building blocks for R1 and R2 groups is also included in Supplementary Materials.

We screened these 127 (70 + 57) mixture samples (25 μM total concentration of each mixture) at 1:100 and 1:1000 dilutions. Several of these mixtures were strongly inhibitory. This provided us with the most active positional R^1^ and R^2^ groups (Figure 3). 

**Figure 4:**
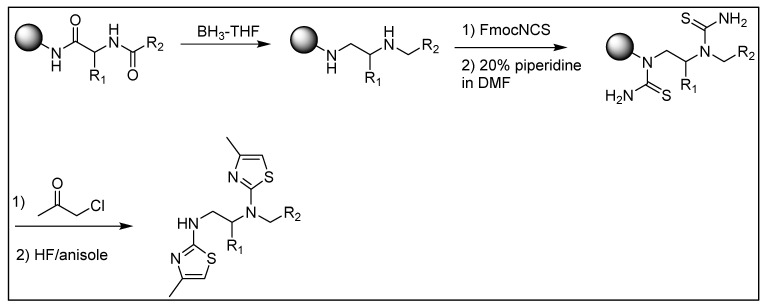
Synthetic strategy of library TPI-2103 and individual compounds TPI-2648.

The deconvolution of the TPI-2103 library led to selection of eight mixtures with defined position R^1^ and thirteen mixtures with defined position R^2^ to prepare a library of individual compounds for testing their anti-adhesion activity. There are seven R^1^ groups in the TPI-2103 sub-library that were associated with inhibition of binding by >50% (Figure 3A). However, R^1^ groups 56 and 36 were not included in the final set as they are the same groups as more active 55 and 18, respectively, but with different chirality, that did not significantly change the activity. Instead, we added groups 17 and 41 to increase diversity among R^1^ groups, and non-active group 51. Similarly, in addition to eleven most active R2 groups (inhibition > 70%), we added modestly active group 73 to explore more diversity for our R^2^ selection and non-active group 81 (Figure 3B). The parallel synthesis of all 104 (8 × 13) individual compounds derived from the deconvolution of TPI-2103 and representing all the combination of selected R^1^ and R^2^ groups was performed by using the strategy outlined in Figure 4. The data show significant differentiation in activity levels among the samples tested at each position, a key feature for deconvoluting a positional scanning library. Moreover, some preliminary SAR can be seen from the library screening. 

Testing individual compounds in vitro and with live infected erythrocytes to select two most active compounds.

**Figure 5:**
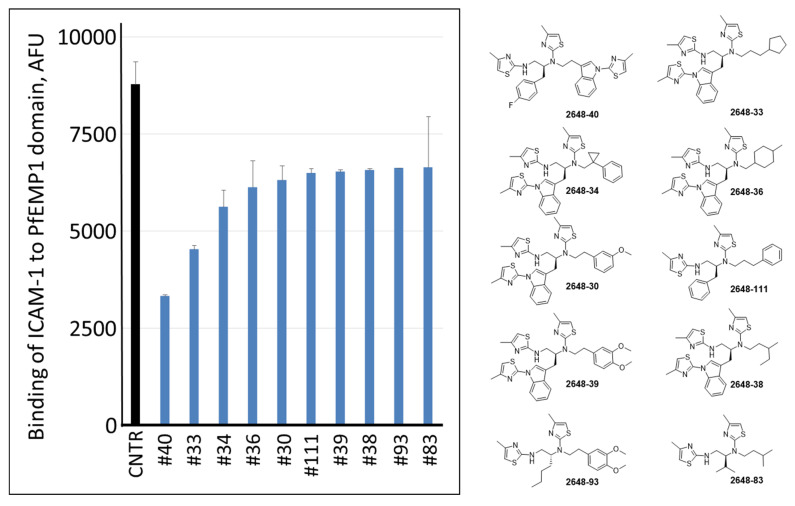
Binding of ICAM-1 to bead-immobilized ICAM-1-binding domain by ten best individual hits of TPI-2648 at 1 μM concentration. Bars represent averages and error bars are SD of two duplicate measurements. CNTR, no drug control. AFU, arbitrary fluorescence unites.

**Figure 6:**
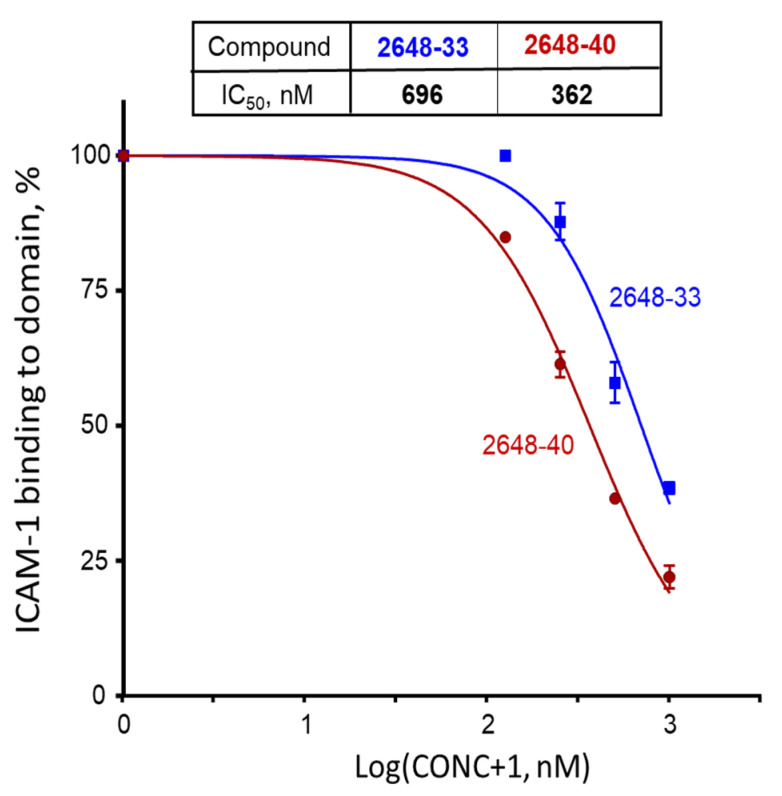
Inhibition of ICAM-1 binding to bead-bound DBL2β3_PF11_0521_ domain by compounds #33 (blue) and #40 (brown). Dots indicate averages and error bars are SEMs of two independent experiments (biological replicates), each performed in duplicate. We measured 100% binding in the absence of compounds, using binding buffer supplemented with the same concentration of DMF as samples with compounds. CONC, compound concentration in nM.

All these 104 individual compounds, named as library TPI-2648 (Supplementary Figures S1 and S2), were tested for inhibition of ICAM-1 binding to bead-immobilized PF11_0521 DBL2β3 domain. In this and following experiments, we kept compounds in the assay all the time (no wash off before addition of ICAM1), to replicate conditions in the next set of experiments on inhibition of binding of live IE to surface-immobilized receptor. Best 10 inhibitory compounds are shown in Figure 5. Interestingly, two most active compounds 2648-40 (or #40) and 2648-33 (or #33) contained R1 and R2 groups that are the 3rd and the 2nd and the 2nd and the 1st, respectively, in a list of activity (Figure 3). Thus, the best activities were observed with compounds having an indole ring containing an extra thiazole at the N position of the indole ring. These two compounds were further characterized for inhibition of ICAM-1 binding to bead-immobilized DBL2β3 domain at different concentrations. Their calculated IC_50_ were 362 nM (95% CI [333–394 nM]) for #40 and 696 nM (95% CI [598–810 nM]) for #33 (Figure 6). However, no reversal of ICAM-1 pre-bound to the domain by these compounds at 1 μM during 3 h was detected, similar to our results with another compound identified previously [31].

**Figure 7:**
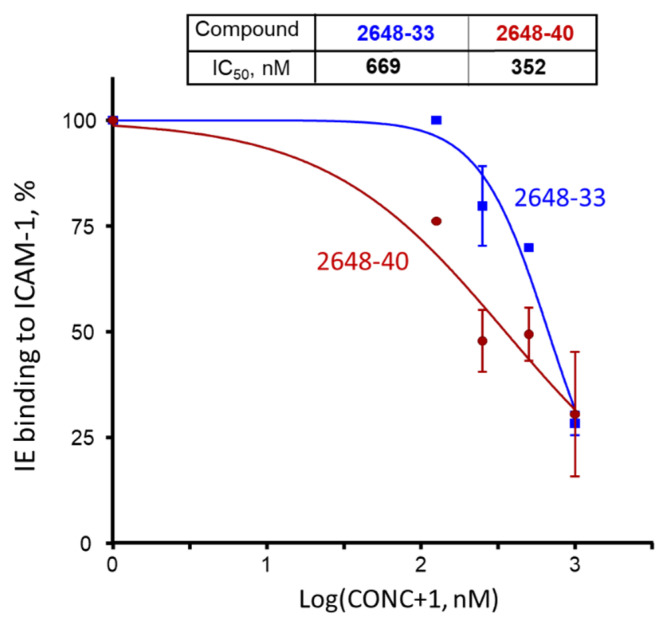
Inhibition of 3G8 line IE binding to surface-immobilized ICAM-1 receptor by compounds #33 (blue) and #40 (brown). Dots indicate averages and error bars are SEMs of bound IE counts in two independent experiments (biological replicates), each performed in duplicate. We measured 100% binding in the absence of compounds, using binding buffer supplemented with the same concentration of DMF as samples with compounds. CONC, compound concentration in nM.

These two compounds were then tested at various concentrations (Figure 7) for inhibition of receptor binding of *heterologous* parasite line 3G8, which binds ICAM-1 [33]. Their calculated IC_50_ values for inhibition of live IE to ICAM-1 receptor were determined as 352 nM (95% CI [163–762 nM]) for #40 and 669 nM (95% CI [524–852 nM]) for #33.

### 2.2. Cytotoxicity Studies of the Two Most Active Compounds 

**Figure 8:**
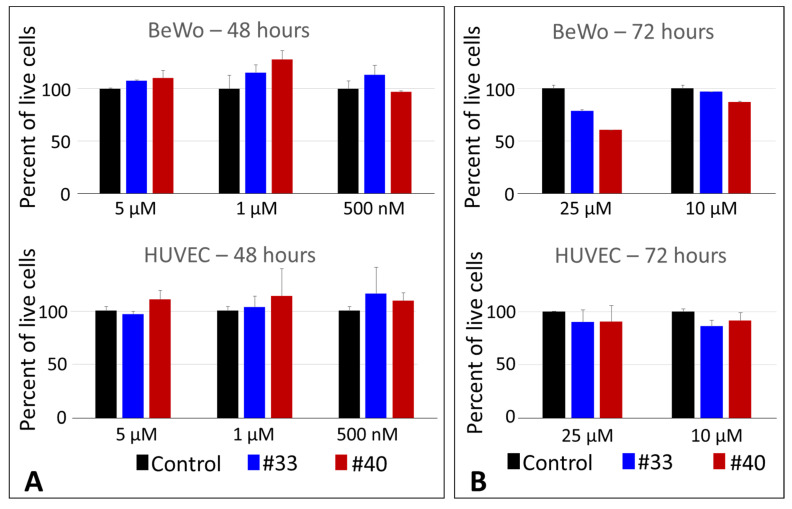
In vitro cellular toxicity of individual compounds #40 and #33 by crystal-violet cell viability assay: (**A**) 48-h assay and (**B**) 72-h assay. Columns represent averages and error bars are SD of duplicate measurements. Data are representative of two independent experiments.

The two compounds with highest ICAM-1 binding inhibition activity 2648-40 and 2648-33 were tested further for cytotoxicity (by crystal violet assay) using two lines of vascular human cells, HUVEC and BeWo. No cytotoxicity was observed at all tested concentrations, up to 5 μM after 48 h for both cell lines (Figure 8A). We then tested higher concentrations (up to 25 μM) and longer time (72 h) and also did not see substantial cytotoxicity (Figure 8B). The 25 μM concentration is ~70 times (compound 2648-40) and ~37 times (compound 2648-33) higher than its respective IC_50_ (for inhibition of binding of live IE to ICAM-1). In addition, no toxicity in 72 h was detected for human erythrocytes at concentrations up to 25 μM (Supplementary Figure S4).

## 3. Discussion

Cytoadherence plays an important role in the lifecycle and virulence of *P. falciparum*, the deadliest of human malaria parasites, and is a cause or prerequisite for SM including placental malaria PM [7] and CM [8,9,10,11]. Moreover, as IE bind to functionally relevant parts of host receptors and inhibit their normal physiological functions [34], anti-adhesion drugs that prevent this interaction would have an additional positive effect on the host. It would be logical to suggest that negating cytoadherence would have a strong positive effect on the host’s fight against infection. Others [35,36] and we believe that anti-adhesion drugs may be very efficient and safe for anti-malarial therapy, used as adjunctive therapy together with parasiticidal drugs.

Due to technical limitations, live malaria parasite isolates of IE are not amenable to high throughput (HT) adaptation for direct screening for anti-adhesion molecules. Our recently developed two-step approach to identify anti-adhesion molecules overcomes these limitations [31]. First, molecules are screened and deconvoluted to single compounds in an HT manner, using inhibition of interactions between known host receptors that are involved in IE adhesion and various PfEMP1 domains that bind these receptors. Second, the molecules identified in the first step are validated for adhesion-inhibitory activity, using live heterologous IE. As the number of hits to test with IE is low, the two-step procedure provides HT identification of anti-adhesion molecules. This published work [31], performed as a proof-of-principle, not only demonstrated the feasibility of the approach, but also identified 3 molecules that inhibit cytoadhesion of CSA-binding IE and 2 molecules that inhibit ICAM-1-binding parasites. However, as library used in that work was of small size, representing only 10,000 Chembridge compounds, the resulting inhibitory molecules obtained had modest potency (IC_50_ in low to mid μM range). In the present work, we screened large size TPI libraries, containing more than 30 million compounds, with the hope to identify more potent molecules.

TPI library collection consists of more than 30 million compounds designed around 75 molecular scaffolds systematically arranged in positional scanning (with at least one fixed position) and scaffold ranking (all variable positions) formats [32,37]. These formats allow for the analysis of millions of compounds by using just hundreds to thousands of samples. The diversity of the TPI lead generation library has been characterized and described quantitatively by means of molecular scaffolds, molecular properties, and structural fingerprints [38]. Fingerprint-based similarity studies demonstrated that individual libraries within the TPI lead generation library occupy highly dense regions in chemical space [38,39]. The high-density coverage increases the potential of identifying activity cliffs [40,41] and provides a rapid understanding of the structure-activity relationship (SAR) associated with novel leads and targets. 

Following the deconvolution of the selected TPI 2103 library, two compounds were identified to inhibit ICAM-1 binding to bead-bound domain at IC_50_ ~ 362 and 696 nM (Figure 6); that is about 50 and ~25 times better than compounds identified in our previous work with small size library [31]. This inhibition activity was similar when using live IE at ~352 and ~669 nM for compounds #40 and #33, respectively (Figure 7). We did not observe any noticeable cytotoxicity at all tested concentrations up to 10 μM for 48 h, and just moderate growth inhibition (21% and 40% for #33 and #40, respectively) only for BeWo cells at 25 μM and 72 h (Figure 8). This concentration is ~70 and ~37 times higher than corresponding IC_50_ values for inhibition of live IE. As these compounds are planned for future development into adjunct emergency treatment, which can be performed by IV injections, there might be not even necessity to use concentrations significantly higher than IC_50_ or IC_100_. Selectivity index (ratio of the 50% cytotoxic concentration (CC_50_) to the 50% inhibition concentration (IC_50_)) of >10 is considered minimally acceptable and >50-fold is an ideal for target candidate profile (TCP-1) [42], as common killing drugs should cross at least 3 membranes for their effect. However, anti-adhesion drugs, injected in the blood, work immediately at the outer surface of IE. We did not determine precise CC_50_ in this work for compounds #40 and #33, but as cytotoxicity was <50% for all tested concentrations, the selectivity index would be better than 70 and 37, which indicates excellent starting profile. 

Another important finding is that better inhibition of ICAM-1 binding on beads (#40 vs. #33) corresponds to better inhibition of binding between IE and surface-immobilized ICAM-1 by these compounds. This is also in accordance with our previous observation in Reference [31]. As our experiments on inhibition of bead-bound domain to ICAM-1 were performed with the domain from 3D7 strain followed by successful inhibition of live IE performed with heterologous strain 3G8, this further strengthen our general approach for high throughput screening procedure for anti-adhesion compounds directed at inhibition of IE sequestration, also in accordance with our previous results [31]. The 3G8 line with IT4 genetic background expresses VAR01 PfEMP1 protein [33], which is encoded by a group C *var* gene and has one ICAM-1-binding DBL2β5 domain with 44% identities, 55% positives, and 15% gaps (by BLAST) compared to 3D7 DBL2β3 ICAM-1-binding domain of PF11_0521 protein encoded by a group A *var* gene in 3D7 line of different genetic background. This level of similarity indicates that interactions between ICAM-1 receptor and quite diverse ICAM-1-binding domains might be inhibited by the same single compound. We believe that our approach might be universally employed for identification of anti-adhesion compounds against various receptor-domain pairs, in spite of a substantial diversity of PfEMP1 domains/proteins. This notion is supported by recent publication [34] describing conserved hydrophobic pocket in PfEMP1 CIDR domains, which allows multiple domain variants bind to conserved CD36 molecule. This pocket might very well be the target (competitive or allosteric) for anti-adhesion molecules providing inhibition of binding of various domains to corresponding receptor and, thus, having broad effects against heterologous lines in the field. Similar structure/functional relationships are feasible for different homologous domain–receptor pairs. Nevertheless, wider spectrum of heterologous parasite lines with specific receptor-binding ability should be tested in experiments on binding inhibition with live IE to confirm our notion. In addition, although certain hydrodynamic forces present during washing steps in plate setup of adhesion inhibition assays used in this work, flow setup might be advantageous to imitate better in vivo conditions of IE cytoadhesion and its blocking/reversal process. These are two limitations of the present study.

Nevertheless, these compounds provide good leads for medicinal chemistry (MC) approaches to develop them into anti-adhesion drugs and test them for prevention of sequestration of ICAM-1-binding parasites that have been implicated into CM and SM.

As was discussed above, binding to ICAM-1 and to EPCR have been implicated into SM and CM. Presence of ICAM-1-binding and EPCR-binding domains combined in a tandem within expressed PfEMP1 molecules in parasites isolated from SM and CM patients was the basis for implicating them into SM or CM [12,27,30,43,44,45,46]. Even if both domains in tandem are required for development of SM and/or CM, it may not necessarily require the use of drugs that inhibit binding of both domains to their corresponding receptors to block IE sequestration. Inhibition of binding to one of them may significantly reduce efficacy of the sequestration of IE. Specifically, for CM, when taking into account the low level of EPCR expression in brain microvasculature, it is logical to suggest that the inhibition of IE binding to ICAM-1, a receptor that is expressed in micro vessels of human brain endothelial cells and is significantly inducible during various brain inflammations (reviewed in Reference [47]), including CM by TNFα (reviewed in Reference [48]), would be sufficient to block sequestration of these IE in the brain and improve the outcome or positively affect the pathology of CM. In this respect, identification of other compounds that will not only prevent but also reverse ICAM-1 sequestration may have significant advantage for anti-adhesion adjunct treatment. As ICAM-1 binds to ICAM-1-binding DBL domains with extremely high avidity down to 2–4 nM [12,22], it might be not a trivial task. Probably, allosteric but not competitive mode of action would be the most usable. As compound leads identified in this work are not able to reverse established ICAM-1–domain interactions (data not shown), this is a limitation of the identified compounds, and molecules with this feature are still needed to be discovered. Nevertheless, just blocking of ICAM-1 binding might be sufficient to reduce or prevent impact of ongoing IE sequestration in SM or CM pathology. This can only be clarified experimentally in future work.

## 4. Conclusions

In this work, we identified, from our screening of the large-size TPIMS library, two small molecules without noticeable cytotoxicity against human cell cultures that can inhibit cytoadhesion of live *P. falciparum* IE to ICAM-1 receptor at IC_50_ down to ~350 nM. This marks significant progress since our previous work, which identified compounds inhibiting ICAM-1 binding in μM range, and a strong foundation for further development of these leads into anti-adhesion drugs for treatment of SM and/or CM complications.

## 5. Materials and Methods

### 5.1. TP-FIU Library of Small Molecules

TP-FIU library collection was described before (as TPIMS library) and consists of more than 30 million compounds in mixtures of various complexity, designed around 75 molecular scaffolds [32,37]. Each mixture in master plate had total concentration of 250 μg/mL in 2.5% dimethylformamide (DMF).

### 5.2. Chemistry General 

^1^H NMR spectra were recorded in DMSO-d6 with TMS as the internal reference solvent for ^1^H NMR (500 MHz) and ^13^C NMR (125 MHz). NMR chemical shifts are expressed in ppm relative to internal solvent peak and coupling constants were calculated in hertz. The reported final purity of the compounds was verified by Shimadzu HPLC and mass spectra under the following conditions: column, Phenomenex Luna 150 × 21.20 mm, 5 micron, C18; mobile phase, (A) H_2_O (+0.1% Formic acid)/(B) MeCN (+0.1% Formic acid), and 3 gradient methods used based on compound hydrophobicity (2% B to 20% B, 11 min) (25% B to 45% B, 31 min) (45% B to 65% B, 21 min); flow rate, 12 mL/min; detection, UV 214 nm. The purity of all final compounds was >95%. All chirality data were generated from the corresponding amino acids. Under the reaction conditions described, no epimerization was observed. Analytical data for active compoundsTPI2648-33 and TPI2648-40 are shown in Supplementary Figure S3.

### 5.3. General Synthesis of the Dithiazole Compounds

All individual TPI-2648 compounds were synthesized following the strategy outlined in Figure 4. The solid-phase synthesis was performed by using the “tea-bag” methodology [49]. Then 100 mg of p-methylbenzdrylamine (pMBHA) resin per compound (1.1 mmol/g, 100–200 mesh) was sealed in a mesh “tea-bag,” neutralized with 5% diisopropylethylamine (DIEA) in dichloromethane (DCM), and subsequently swelled with additional DCM washes. The first diversity R^1^ was introduced by the coupling of Boc amino acid (6 eq.) in dimethylformamide (0.1 M DMF) for 60 min in the presence of diisopropylcarbodiimide (DIC, 6 equiv.) and 1-hydroxybenzotriazole hydrate (HOBt, 6 equiv.). The Boc protecting group was removed with 55% TFA/DCM for 30 min and subsequently neutralized 3 times with 5% DIEA/DCM. The second diversity (R^2^) was introduced by the subsequent coupling of a carboxylic (10 eq.) in the presence of DIC (10 eq.) overnight in DMF anhydrous. All coupling reactions were monitored for completion by Ninhydrin test.

The reduction of the amide bonds was performed in a 1000 mL Wilmad LabGlass vessel under nitrogen in the presence of 1.0M Borane–Tetrahydrofuran (BH_3_–THF) complex solution (40-fold excess of BH_3_–THF per amide bond). The reaction vessel was heated to 65 °C, and the temperature was maintained for 72 h. [50,51] The solution was then discarded and the bags were washed with THF and methanol. Once completely dry, the bags were treated overnight with piperidine at 65 °C and washed several times with methanol, DMF and DCM. Before proceeding, completion of the reduction was monitored by a control cleavage and analyzed by LCMS.

The generated resin bound diamines were treated with Fmoc isothiocyanate (10 eq.) in anhydrous DMF overnight. The Fmoc protecting group was released by piperidine in DMF (20:80) and the generated thioureas were treated with Chloroacetone (10 eq.) in DMSO in the presence of DIEA (10 eq.) overnight at 65 °C. The desired dithiazole compounds were cleaved from the solid-support in the presence of HF and then extracted with acetic acid and lyophilized to obtain solid white powder. The obtained white powders were purified by using preparative high-performance liquid chromatography and the desired compounds were obtained in good yields and high purity (>95%).

Library Screening to identify molecules that inhibit ICAM-1 binding to PF11_0521 DBL2β3 domain Immobilized on BioPlex Beads was performed as described in details before [31]. Briefly, all samples were tested in duplicates and were diluted in binding buffer (PBS with 0.05% tween-20 and 0.1% Bovine Serum Albumin (BSA)), with controls of equivalent DMF concentrations. All incubations were conducted at room temperature. BioPlex Beads with surface-immobilized ICAM-1-binding domain DBL2β3 from PF11_0521 PfEMP1 (3D7 line genetic background) or an empty control construct HisAdEx were used to test for inhibition of ICAM-1 binding. Then 96-well MultiScreen filter plates (Millipore) were blocked with 200 µL of binding buffer for 1 h at room temperature. The wells were then vacuumed and 50 µL of bead mix (two bead regions, with immobilized domain and control construct) resuspended in binding buffer were added, vacuumed, and followed by addition of 50 µL of small molecules and incubation on a shaker at approximately 600 rpm for 30 min. For screening of the initial 75-scaffold library and for deconvolution of the PTI-2103 library, compounds were washed off by binding buffer, then 50 µL of biotinylated ICAM-1 receptors were added to the samples for a final concentration of 0.5 µg/mL, followed by another incubation for 1 h on the shaker. For screening compounds from the individual compound library TPI-2648, we omitted wash of the compounds and added ICAM-1 receptor directly to the mixture of beads and drugs to the same final concentration of 0.5 µg/mL, followed by another incubation for 1 h on the shaker. The plate was then vacuumed and washed with 150 µL of PBS plus 0.05% tween-20 (1XPBST) two times and 75 µL of streptavidin–phycoerythrin (Jackson Immunoresearch, West Grove, PA, USA) at 1:250 was added and incubated for another hour on the shaker. The samples were then washed with 150 µL of 1XPBST two times, followed by one wash with 150 µL 1XPBS, and then resuspended in 125 µL of 1XPBS and incubated on the shaker for 5 min at 1100 rpm before being placed on BioPlex 200 (BioRad, Hercules, CA, USA) for analysis. In a typical experiment, binding levels of soluble ICAM-1 receptor to the bead-immobilized DBL domain were several thousand counts measured in arbitrary fluorescence units (AFU) and up to 100 AFU to control construct HisAdEx. Difference between DBL domain binding and HisAdEx control defines 100% binding. Pre-incubation of ICAM-1 with monoclonal antibody (mAb) My-13 (Abcam, Cambridge, UK) leads to ~90% reduction of ICAM-1 binding to DBL domain, serving as a control for efficient inhibition of binding in the experiment.

### 5.4. Reversal of ICAM-1 Binding to PF11_0521 DBL2β3 Domain

Reversal of receptor binding was performed by incubation of bead-immobilized domains with ICAM-1, washing away unbound receptor, incubating with selected compounds at 1 μM concentration for 3 h, and detecting bound receptor, as previously described [17,31].

### 5.5. Parasite Culture

Human O+ blood and AB serum (HP1022) for growth of *P. falciparum* parasites was purchased from Valley Biomedical. Parasite cultures ware maintained in O+ blood (Valley Biomedical) in complete RPMI at 37 °C in 5% O_2_, 5% CO_2_, and 90% N_2_ gas mixture and had 2% hematocrit and 3–8% parasitemia.

Inhibition of 3G8 IE Binding to ICAM-1 receptor by selected compounds was performed as described in detail in References [31,52]. Briefly, ICAM-1 receptor (or BSA as a control) was spotted on a plastic petri dish at 10 µg/mL and incubated at 4 °C, overnight. After overnight incubation, any unattached receptors were removed by washing with PBS, and spots were blocked by 3% BSA in 1XPBS at 37 °C for thirty minutes. PBS–BSA was removed and plate washed with 1XPBS twice. Before the adhesion assays, IE in trophozoite stage were enriched by magnetic LD columns (Miltenyi Biotec, Bergisch Gladbach, Germany, cat#130-042-901) as described by manufacturer. Percentage of mature trophozoites in the elution was adjusted to desired parasitemia level, using uninfected erythrocytes. The erythrocytes infected with the 3G8 parasite line (IT4 genetic background, kindly provided by Dr. Joe Smith) that bind ICAM-1 [33] at approximately 30% parasitemia were incubated with compounds for 30 min at 37 °C before being applied to the plastic Petri dish–immobilized receptor. The final concentration of the solvent (DMF or DMSO), also used as a no-treatment control, in all experiments, was at or below 0.1%. The parasites were then applied to the receptor spots and incubated at 37 °C, for thirty minutes, on a shaker, at 50 rpm, and then they were gently washed from the unbound IEs, by 1XPBS, on a shaker. A total of 1.5% glutaraldehyde in 1X PBS was applied for 15 min, at room temperature, to fix the attached IEs, followed by incubation with 10% Giemsa stain, for 15 min, at room temperature. The plate was then washed with deionized water, dried overnight, and counted (≥10+ microscope fields per spot, each spot in duplicate) under the microscope, at 1000X magnification, using an oil immersion lens. Averaged counts in BSA spots were subtracted from averaged counts in spots with receptor (tested without or with drugs). Difference between receptor spot (without drug) and BSA control defines 100% binding (typically, between 800 and 2000 IE per mm^2^). 

### 5.6. Crystal Violet Cell Viability Assay

Two human cell lines were used in these experiments: BeWo and HUVEC (both from ATCC). Cells were grown as described by ATCC protocols. Cell suspension at 5 × 10^4^ cells/mL were prepared and approximately 5 × 10^3^ cells were seeded into a 96-well flat bottom cell culture plate. Compounds at various concentrations were diluted in 1XPBS. No-treatment controls contained equal concentrations of DMF, and not more than 0.25% (at highest compound concentration of 25 μM). Cells were incubated for 48 or 72 h at 5% CO_2_ and 37 °C. After the incubation period, the cell growth media along with any dead cells were pipetted out and cells were then washed with 1XPBS and fixed with 100 µL of 3.7% formaldehyde (Sigma Life Science, Burlington, USA) for 30 min. Cells were then stained with 100 µL of 0.1% crystal violet (Sigma Life Science, Crystal Violet Solution 1%, Aqueous Solution) for 10 min, washed abundantly with deionized water, and allowed to dry at room temperature. Then 100 µL of 10% acetic acid (Fisher Scientific, Waltham, MA, USA, BP2401-500) was added, and absorbance of samples was measured on a spectrophotometer at 595 nm.

Cytotoxicity of compounds for RBC was tested as described previously [53,54]. Briefly, RBC (O+) at 2% hematocrit were seeded in 96-well plates and incubated with compounds at 1, 5, 10, and 25 μM concentrations for 72 h in duplicates. RBC incubated with complete RPMI supplemented with 0.1% Triton X-100 were used as a positive cytotoxic control. At 72 h, the medium was removed and tested, using the lactate dehydrogenase (LDH) cytotoxicity kit (Thermo Fisher Scientific, Waltham, MA, USA). The results of cytotoxicity assay were calculated as described in the kit manual.

### 5.7. Statistical Analyses

GraphPad Prizm 6 software was used. Inhibition of mixtures or individual compounds relative to non-inhibited control was analyzed by one-way ANOVA, using Dunnett’s multiple comparisons test. IC_50_ values were determined by non-linear regression (fitted by least square) of dose–response/inhibition curves (Log(inhibitor concentration, nM) vs. normalized response—variable slope). To include “0” concentration of compounds on these graphs illustrating binding inhibition, we used Log(Inhibitor concentration+1, nM) scale for the *x*-axis.

## Data Availability

All data generated or analyzed during this study are included in this article (and its Supplementary Materials files).

## Supplementary Materials

The following are available online at https://www.mdpi.com/article/10.3390/ijms22115659/s1. Title: Small molecule compounds, identified from mixture-based library, to inhibit binding between Plasmodium falciparum infected erythrocytes and endothelial receptor ICAM-1. Supplementary Figure S1. R^1^ and R^2^ distribution for the positional scanning library TPI-2103. Supplementary Figure S2: Individual compounds derived from the deconvolution of library TPI-2103: synthesis of individual compounds TPI-2648. Supplementary Figure S3: Analytical data for active compoundsTPI2648-33 and TPI2648-40. Supplementary Figure S4: Cytotoxic effect of compounds 2648-33 and 2648-40 on human erythrocytes. Compounds tested at shown concentrations (Conc.). Positive control is 0.1 % Triton X-100. Negative controls are PBS and 0.25% DMF. Bars are averages of duplicates and error bars are Standard Deviations.
