# Supplementary material for: Small Molecule Compounds Identified from Mixture-Based Library Inhibit Binding between Plasmodium falciparum Infected Erythrocytes and Endothelial Receptor ICAM-1
Source: Int J Mol Sci. 2021 May 26;22(11):5659. doi: 10.3390/ijms22115659 (PMC8198633; doi:10.3390/ijms22115659)

Small molecule compounds, identified from mixture-based library, to inhibit binding between *Plasmodium falciparum* infected erythrocytes and endothelial receptor ICAM-1

Olga Chesnokov[1#], Pimnitah Visitdesotrakul[1#], Komal Kalani[2], Adel Nefzi[2*], and Andrew V. Oleinikov[1*]

[1] Charles E. Schmidt College of Medicine, Florida Atlantic University, Boca Raton, FL

[2] Florida International University (FIU), Miami, Florida

Supplementary Materials

Supplementary Figures S1 – S4

Supplementary Figure S1. R1 and R2 distribution for the positional scanning library TPI-2103. Groups selected for synthesis of TPI-2648 library (Supplementary Figure 2) are indicated by yellow highlights.

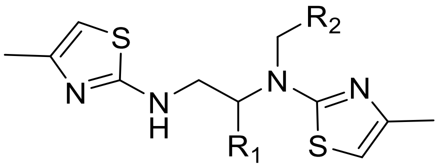

2103	R_1 (derived from the amino acid side chain)	R_2 (corresponding carboxylic acid)
2103-1	Boc-L-Ala-OH	X
2103-2	Boc-L-Asp(OBzl)-OH	X
2103-3	Boc-L-Glu(OBzl)-OH	X
2103-4	Boc-L-Phe-OH	X
2103-5	Boc-Gly-OH	X
2103-6	Boc-L-Ile-OH	X
2103-7	Boc-L-His(Tos)-OH	X
2103-8	Boc-L-Lys(ClZ)-OH	X
2103-9	Boc-L-Leu-OH	X
2103-10	Boc-L-Met(O)-OH	X
2103-11	Boc-L-Asn-OH	X
2103-12	Boc-L-Pro-OH	X
2103-13	Boc-L-Gln-OH	X
2103-14	Boc-L-Arg(Tos)-OH	X
2103-15	Boc-L-Ser(Bzl)-OH	X
2103-16	Boc-L-Thr(Bzl)-OH	X
2103-17	Boc-L-Val-OH	X
2103-18	Boc-L-Trp-OH	X
2103-19	Boc-L-Tyr(2-Br-Z)-OH	X
2103-20	Boc-D-Ala-OH	X
2103-21	Boc-D-Asp(OBzl)-OH	X
2103-22	Boc-D-Glu(OBzl)-OH	X
2103-23	Boc-D-Phe-OH	X
2103-24	Boc-D-His(Tos)-OH	X
2103-25	Boc-D-Ile-OH	X
2103-26	Boc-D-Lys(ClZ)-OH	X
2103-27	Boc-D-Leu-OH	X
2103-28	Boc-D-Met(O)-OH	X
2103-29	Boc-D-Asn-OH	X
2103-30	Boc-D-Pro-OH	X
2103-31	Boc-D-Gln-OH	X
2103-32	Boc-D-Arg(Tos)-OH	X
2103-33	Boc-D-Ser(Bzl)-OH	X
2103-34	Boc-D-Thr(Bzl)-OH	X
2103-35	Boc-D-Val-OH	X
2103-36	Boc-D-Trp-OH	X
2103-37	Boc-D-Tyr(2-Br-Z)-OH	X
2103-38	Boc-L-Aib-OH	X
2103-39	Boc-D-Nva-OH	X
2103-40	Boc-L-Nle-OH	X
2103-41	Boc-D-Nle-OH	X
2103-42	Boc-L-Orn(ClZ)-OH	X
2103-43	Boc-D-Orn(ClZ)-OH	X

2103-44	Boc-L-Phg-OH	X
2103-45	Boc-D-Phg-OH	X
2103-46	Boc-L-2-Naphtylala-OH	X
2103-47	Boc-D-2-Naphtylala-OH	X
2103-48	Boc-b-Ala-OH	X
2103-49	Boc-L-Cha-OH	X
2103-50	Boc-D-Cha-OH	X
2103-51	Boc-L-p-Nitro-Phenylalanine	X
2103-52	Boc-D-p-Nitro-Phenylalanine	X
2103-53	Boc-L-4-Chlorophenylalanine	X
2103-54	Boc-D-4-Chlorophenylalanine	X
2103-55	Boc-L-4-Fluorophenylalanine	X
2103-56	Boc-D-4-Fluorophenylalanine	X
2103-57	Boc-3,4-dehydro-L-Proline	X
2103-58	X	1-phenyl-1-cyclopropanecarboxylic acid
2103-59	X	2-Phenylbutyric Acid
2103-60	X	3-Phenylbutyric Acid
2103-61	X	m-Tolylacetic acid
2103-62	X	3-Fluorophenylacetic Acid
2103-63	X	3-Bromophenylacetic Acid
2103-64	X	p-Tolyacetic acid
2103-65	X	4-Fluorophenylacetic acid
2103-66	X	3-Methoxyphenylacetic acid
2103-67	X	4-Bromophenylacetic acid
2103-68	X	4-Methoxyphenylacetic acid
2103-69	X	3,4-Dimethoxyphenyl acetic acid
2103-70	X	4-isobutyl-alpha-Methylphenylacetic Acid
2103-71	X	3,4-Dichlorophenylacetic acid
2103-72	X	3,5-Bis(Trifluoromethyl)-Phenylacetic acid
2103-73	X	3-(3,4-Dimethoxyphenyl)-propionic Acid
2103-74	X	Phenylacetic acid
2103-75	X	3,4,5-Trimethoxybenzoic acid
2103-76	X	Butyric Acid
2103-77	X	Heptanoic Acid
2103-78	X	Isobutyric Acid
2103-79	X	2-Methylbutiric Acid
2103-80	X	Isovaleric acid
2103-81	X	3-Methylvaleric acid
2103-82	X	p-Toluic Acid
2103-83	X	cyclopentanecarboxylic acid.
2103-84	X	cyclohexanecarboxilic acid
2103-85	X	cyclohexylacetic acid
2103-86	X	cyclohexanebutyric acid
2103-87	X	cycloheptanecarboxylic acid
2103-88	X	1-Adamantaneacetic Acid
2103-89	X	cyclobutanecarboxylic acid
2103-90	X	3-cyclopentylpropionic acid
2103-91	X	cyclohexanepropionic acid
2103-92	X	4-methyl-1-cyclohexancarboxylic acid
2103-93	X	4-tert-butyl-cyclohexancecarboxylic acid
2103-94	X	4-biphenylacetic acid
2103-95	X	1-Adamantancecarboxylic acid
2103-96	X	4-Methylvaleric acid

2103-97	X	2-norbornaneacetic acid
2103-98	X	Hexanoic Acid
2103-99	X	Octanoic Acid
2103-100	X	2-Ethylbutyric Acid
2103-101	X	Trimethylacetic Acid
2103-102	X	Cyclopentylacetic Acid
2103-103	X	3-Cyclopentylpropionic Acid
2103-104	X	2-ethylhexanoic acid
2103-105	X	2-Phenoxypropionic acid
2103-106	X	Benzoic acid
2103-107	X	2-Chlorobenzoic acid
2103-108	X	2-(P-Toluoyl)- Benzoic acid
2103-109	X	m-Toluic acid
2103-110	X	4-FlouroBenzoic Acid
2103-111	X	4-Bromobenzoic Acid
2103-112	X	4-Ethylbiphenyl-4'-carboxylic acid
2103-113	X	3,4-Dimethylbenzoic acid
2103-114	X	4-Biphenylcarboxylic Acid
2103-115	X	2-BenzoylBenzoic acid
2103-116	X	1-Naphthoic acid
2103-117	X	2-Furoic acid
2103-118	X	Indole-3-acetic acid
2103-119	X	cyclohexanepropionic acid
2103-120	X	3,3-diphenylpropionic acid
2103-121	X	5-Methyl-2-pyrazinecarboxylic acid
2103-122	X	2-Benzimidazolepropionic acid
2103-123	X	4-Phenylbutyric acid
2103-124	X	5-Bromo-2-furoic acid
2103-125	X	3-Bromopropionic acid
2103-126	X	3,3,3-triphenylpropionic acid
2103-127	X	Myristic Acid

Supplementary Figure S2. Individual compounds derived from the deconvolution of library TPI-2103: synthesis of individual compounds TPI-2648

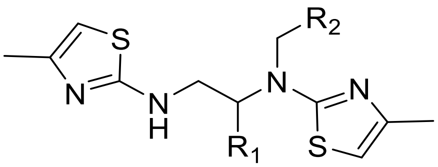

	**R_1 (derived from the amino acid side chain)**	**R_2 (corresponding carboxylic acid)**
1	Boc-L-Thr(Bzl)-OH	Indole-3-acetic acid
2	Boc-L-Thr(Bzl)-OH	3,4-dimethoxyphenylacetic acid
3	Boc-L-Thr(Bzl)-OH	heptanoic acid
4	Boc-L-Thr(Bzl)-OH	3-methoxyphenylacetic acid
5	Boc-L-Thr(Bzl)-OH	isovaleric acid
6	Boc-L-Thr(Bzl)-OH	2-methylbutyric acid
7	Boc-L-Thr(Bzl)-OH	3-cyclopentylpropionic acid
8	Boc-L-Thr(Bzl)-OH	1-phenyl-1-cyclohexanecarboxylc acid
9	Boc-L-Thr(Bzl)-OH	3-phenylbutyric acid
10	Boc-L-Thr(Bzl)-OH	4-methyl-1-cyclohexanecarboxylic acid
11	Boc-L-Thr(Bzl)-OH	3,5-bis(trifluoromethyl)-phenylacetic acid
12	Boc-L-Thr(Bzl)-OH	3-methylvaleric acid
13	Boc-L-Thr(Bzl)-OH	3-(3,4-dimethoxyphenyl)propionic acid
14	Boc-L-4-Chlorophenylalanine	Indole-3-acetic acid
15	Boc-L-4-Chlorophenylalanine	3,4-dimethoxyphenylacetic acid
16	Boc-L-4-Chlorophenylalanine	heptanoic acid
17	Boc-L-4-Chlorophenylalanine	3-methoxyphenylacetic acid
18	Boc-L-4-Chlorophenylalanine	isovaleric acid
19	Boc-L-4-Chlorophenylalanine	2-methylbutyric acid
20	Boc-L-4-Chlorophenylalanine	3-cyclopentylpropionic acid
21	Boc-L-4-Chlorophenylalanine	1-phenyl-1-cyclohexanecarboxylc acid
22	Boc-L-4-Chlorophenylalanine	3-phenylbutyric acid
23	Boc-L-4-Chlorophenylalanine	4-methyl-1-cyclohexanecarboxylic acid
24	Boc-L-4-Chlorophenylalanine	3,5-bis(trifluoromethyl)-phenylacetic acid
25	Boc-L-4-Chlorophenylalanine	3-methylvaleric acid
26	Boc-L-4-Chlorophenylalanine	3-(3,4-dimethoxyphenyl)propionic acid
27	Boc-L-Trp-OH	Indole-3-acetic acid
28	Boc-L-Trp-OH	3,4-dimethoxyphenylacetic acid
29	Boc-L-Trp-OH	heptanoic acid
30	Boc-L-Trp-OH	3-methoxyphenylacetic acid
31	Boc-L-Trp-OH	isovaleric acid
32	Boc-L-Trp-OH	2-methylbutyric acid
33	Boc-L-Trp-OH	3-cyclopentylpropionic acid
34	Boc-L-Trp-OH	1-phenyl-1-cyclohexanecarboxylc acid
35	Boc-L-Trp-OH	3-phenylbutyric acid
36	Boc-L-Trp-OH	4-methyl-1-cyclohexanecarboxylic acid

37	Boc-L-Trp-OH	3,5-bis(trifluoromethyl)-phenylacetic acid
38	Boc-L-Trp-OH	3-methylvaleric acid
39	Boc-L-Trp-OH	3-(3,4-dimethoxyphenyl)propionic acid
40	Boc-L-4-fluoroPhenylalanine	Indole-3-acetic acid
41	Boc-L-4-fluoroPhenylalanine	3,4-dimethoxyphenylacetic acid
42	Boc-L-4-fluoroPhenylalanine	heptanoic acid
43	Boc-L-4-fluoroPhenylalanine	3-methoxyphenylacetic acid
44	Boc-L-4-fluoroPhenylalanine	isovaleric acid
45	Boc-L-4-fluoroPhenylalanine	2-methylbutyric acid
46	Boc-L-4-fluoroPhenylalanine	3-cyclopentylpropionic acid
47	Boc-L-4-fluoroPhenylalanine	1-phenyl-1-cyclohexanecarboxylc acid
48	Boc-L-4-fluoroPhenylalanine	3-phenylbutyric acid
49	Boc-L-4-fluoroPhenylalanine	4-methyl-1-cyclohexanecarboxylic acid
50	Boc-L-4-fluoroPhenylalanine	3,5-bis(trifluoromethyl)-phenylacetic acid
51	Boc-L-4-fluoroPhenylalanine	3-methylvaleric acid
52	Boc-L-4-fluoroPhenylalanine	3-(3,4-dimethoxyphenyl)propionic acid
53	Boc-L-Glu(Obzl)-OH	Indole-3-acetic acid
54	Boc-L-Glu(Obzl)-OH	3,4-dimethoxyphenylacetic acid
55	Boc-L-Glu(Obzl)-OH	heptanoic acid
56	Boc-L-Glu(Obzl)-OH	3-methoxyphenylacetic acid
57	Boc-L-Glu(Obzl)-OH	isovaleric acid
58	Boc-L-Glu(Obzl)-OH	2-methylbutyric acid
59	Boc-L-Glu(Obzl)-OH	3-cyclopentylpropionic acid
60	Boc-L-Glu(Obzl)-OH	1-phenyl-1-cyclohexanecarboxylc acid
61	Boc-L-Glu(Obzl)-OH	3-phenylbutyric acid
62	Boc-L-Glu(Obzl)-OH	4-methyl-1-cyclohexanecarboxylic acid
63	Boc-L-Glu(Obzl)-OH	3,5-bis(trifluoromethyl)-phenylacetic acid
64	Boc-L-Glu(Obzl)-OH	3-methylvaleric acid
65	Boc-L-Glu(Obzl)-OH	3-(3,4-dimethoxyphenyl)propionic acid
66	Boc-L-4-nitrophenylalanine	Indole-3-acetic acid
67	Boc-L-4-nitrophenylalanine	3,4-dimethoxyphenylacetic acid
68	Boc-L-4-nitrophenylalanine	heptanoic acid
69	Boc-L-4-nitrophenylalanine	3-methoxyphenylacetic acid
70	Boc-L-4-nitrophenylalanine	isovaleric acid
71	Boc-L-4-nitrophenylalanine	2-methylbutyric acid
72	Boc-L-4-nitrophenylalanine	3-cyclopentylpropionic acid
73	Boc-L-4-nitrophenylalanine	1-phenyl-1-cyclohexanecarboxylc acid
74	Boc-L-4-nitrophenylalanine	3-phenylbutyric acid
75	Boc-L-4-nitrophenylalanine	4-methyl-1-cyclohexanecarboxylic acid
76	Boc-L-4-nitrophenylalanine	3,5-bis(trifluoromethyl)-phenylacetic acid
77	Boc-L-4-nitrophenylalanine	3-methylvaleric acid
78	Boc-L-4-nitrophenylalanine	3-(3,4-dimethoxyphenyl)propionic acid
79	Boc-L-Val-OH	Indole-3-acetic acid
80	Boc-L-Val-OH	3,4-dimethoxyphenylacetic acid
81	Boc-L-Val-OH	heptanoic acid
82	Boc-L-Val-OH	3-methoxyphenylacetic acid

83	Boc-L-Val-OH	isovaleric acid
84	Boc-L-Val-OH	2-methylbutyric acid
85	Boc-L-Val-OH	3-cyclopentylpropionic acid
86	Boc-L-Val-OH	1-phenyl-1-cyclohexanecarboxylc acid
87	Boc-L-Val-OH	3-phenylbutyric acid
88	Boc-L-Val-OH	4-methyl-1-cyclohexanecarboxylic acid
89	Boc-L-Val-OH	3,5-bis(trifluoromethyl)-phenylacetic acid
90	Boc-L-Val-OH	3-methylvaleric acid
91	Boc-L-Val-OH	3-(3,4-dimethoxyphenyl)propionic acid
92	Boc-D-Nle-OH	Indole-3-acetic acid
93	Boc-D-Nle-OH	3,4-dimethoxyphenylacetic acid
94	Boc-D-Nle-OH	heptanoic acid
95	Boc-D-Nle-OH	3-methoxyphenylacetic acid
96	Boc-D-Nle-OH	isovaleric acid
97	Boc-D-Nle-OH	2-methylbutyric acid
98	Boc-D-Nle-OH	3-cyclopentylpropionic acid
99	Boc-D-Nle-OH	1-phenyl-1-cyclohexanecarboxylc acid
100	Boc-D-Nle-OH	3-phenylbutyric acid
101	Boc-D-Nle-OH	4-methyl-1-cyclohexanecarboxylic acid
102	Boc-D-Nle-OH	3,5-bis(trifluoromethyl)-phenylacetic acid
103	Boc-D-Nle-OH	3-methylvaleric acid
104	Boc-D-Nle-OH	3-(3,4-dimethoxyphenyl)propionic acid

Supplementary Figure S3. Analytical data for active compoundsTPI2648-33 and TPI2648-40

Compound 2648-40: ^{1}H NMR (500 MHz, DMSO-d_6) δppm 10.89 (bs, 1H), 8.29 (d, J= 8.96 Hz, 1H), 8.28 (d, J= 8.96 Hz, 1H), 7.71 (m, 2H), 7.28 (m, 3H), 7.11 (m, 3H), 6.35 (s, 1H), 6.33 (s, 1H), 6.32 (s, 1H), 2.86-3.02 (m, 7H), 2.40 (s, 3H), 2.39 (s, 3H), 2.14 (s, 3H), 1.90-1.91 (m, 2H).

MS (ESI): m/z calcd for $C_{31}H_{31}FN_6S_3$ $[M + H]^+$: 603.2, found: 603.0.

Compound 2648-33: ^{1}H NMR (500 MHz, DMSO-d_6) δppm 10.93 (s, 1H), 8.18 (m, 1H), 7.74 (m, 2H), 7.32 (m, 1H), 7.21 (s, 1H), 7.11 (s, 1H), 7.01 (s, 1H), 6.53 (s, 1H), 2.89-3.30 (m, 5H), 2.31-2.36 (m, 2H), 2.31 (s, 6H), 2.08-2.15 (m, 2h), 1.91 (s, 3H), 1.23-1.64 (m, 11H).

MS (ESI): m/z calcd for $C_{31}H_{38}N_6S_3$ $[M + H]^+$: 591.2, found: 591.0.

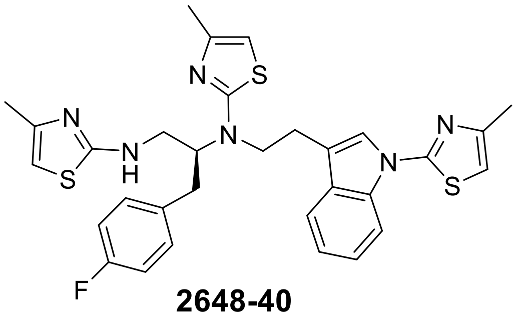

2648-40

Chemical Formula: $C_{31}H_{31}FN_6S_3$
Exact Mass: 602.18
Molecular Weight: 602.81

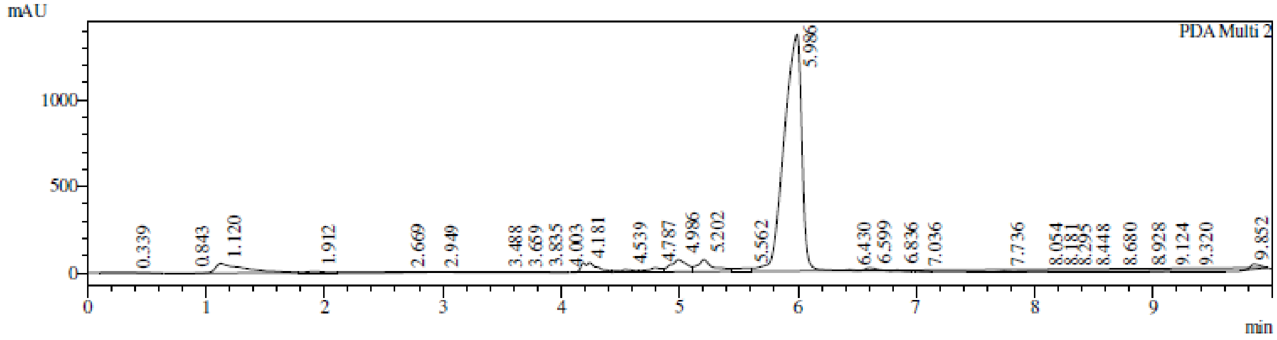

Peak#:4 Ret.Time:Averaged 6.063-6.070(Scan#:1820-1822)
BG Mode:Calc 5.693<->6.587(1709<->1977)
Mass Peaks:51 Base Peak:603.05(16408351) Polarity:Pos Segment1 - Event1

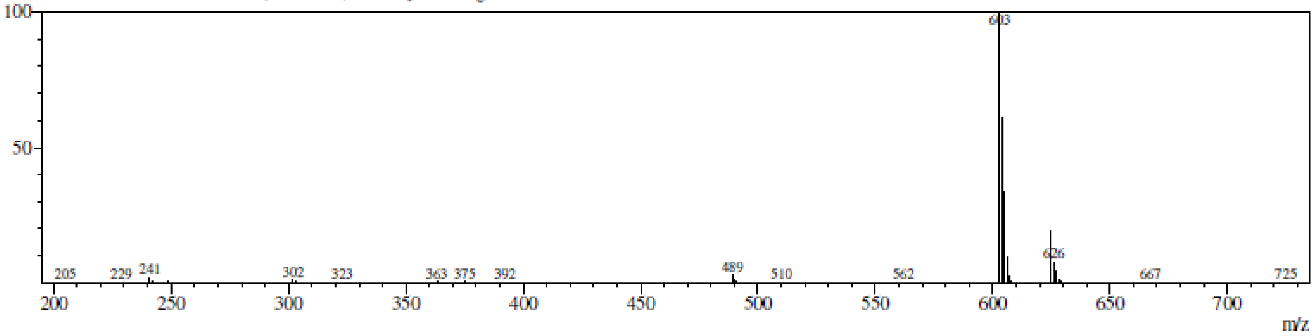

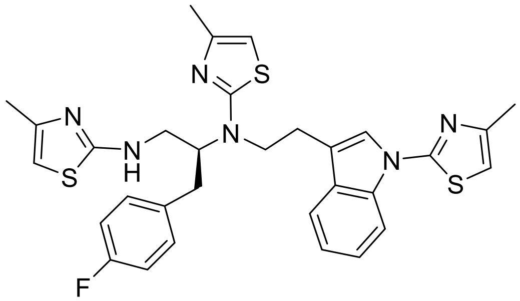

2648-40

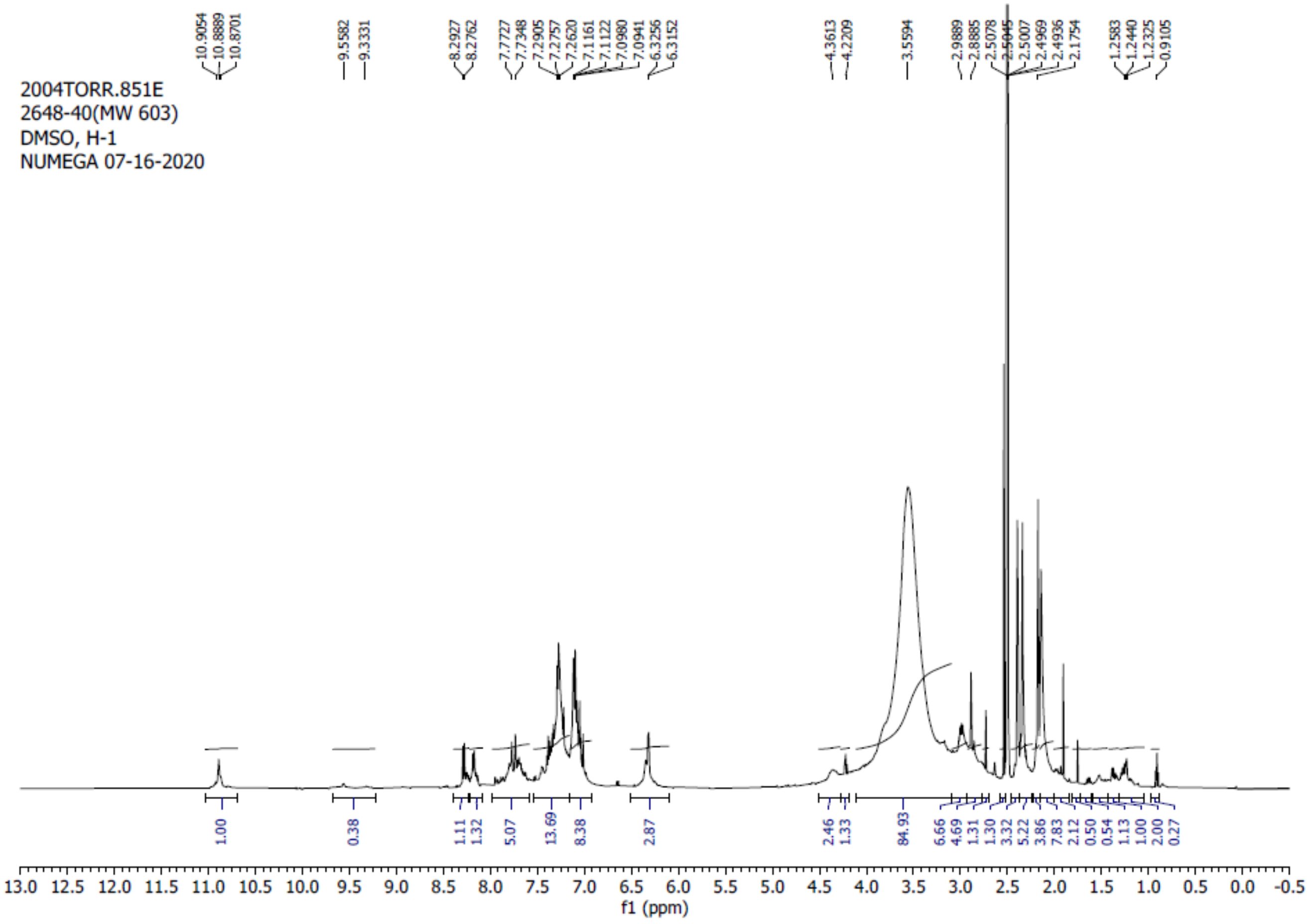

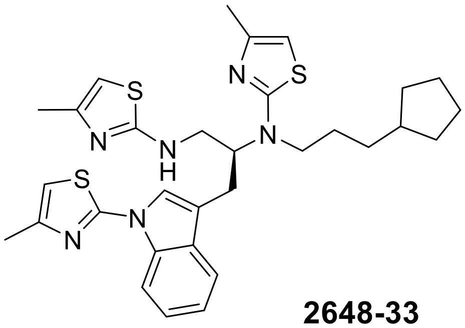

2648-33

Chemical Formula: $C_{31}H_{38}N_6S_3$
Exact Mass: 590.23
Molecular Weight: 590.87

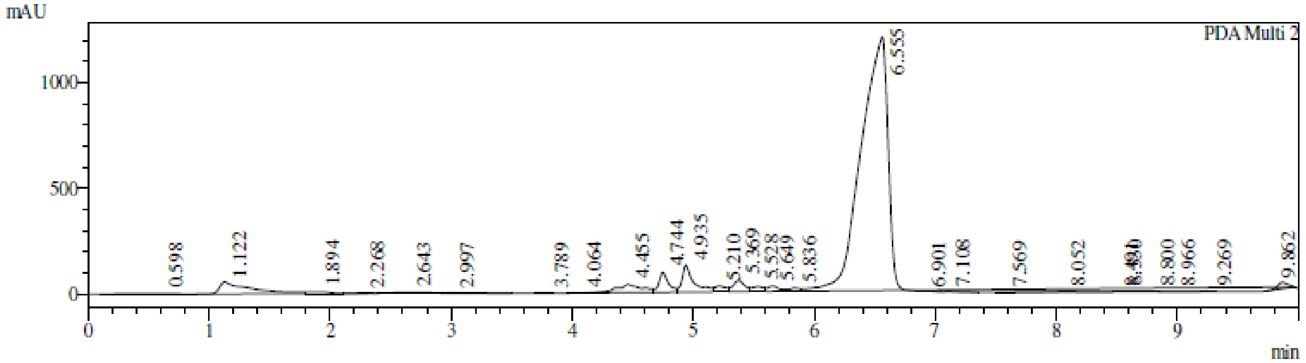

Peak#:5 Ret.Time:Averaged 6.617-6.623(Scan#:1986-1988)
BG Mode:Calc 6.080<->6.940(1825<->2083)
Mass Peaks:72 Base Peak:591.10(13088246) Polarity:Pos Segment1 - Event1

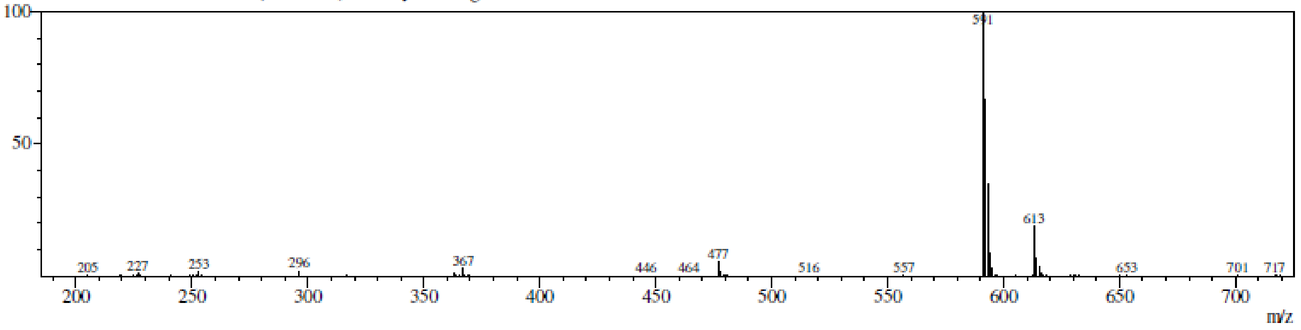

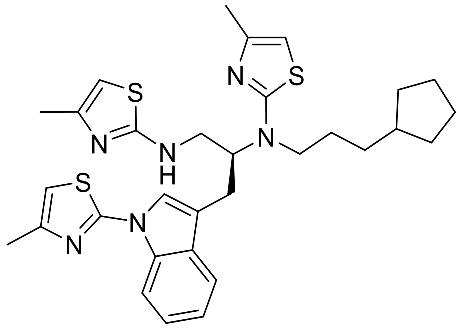

2648-33

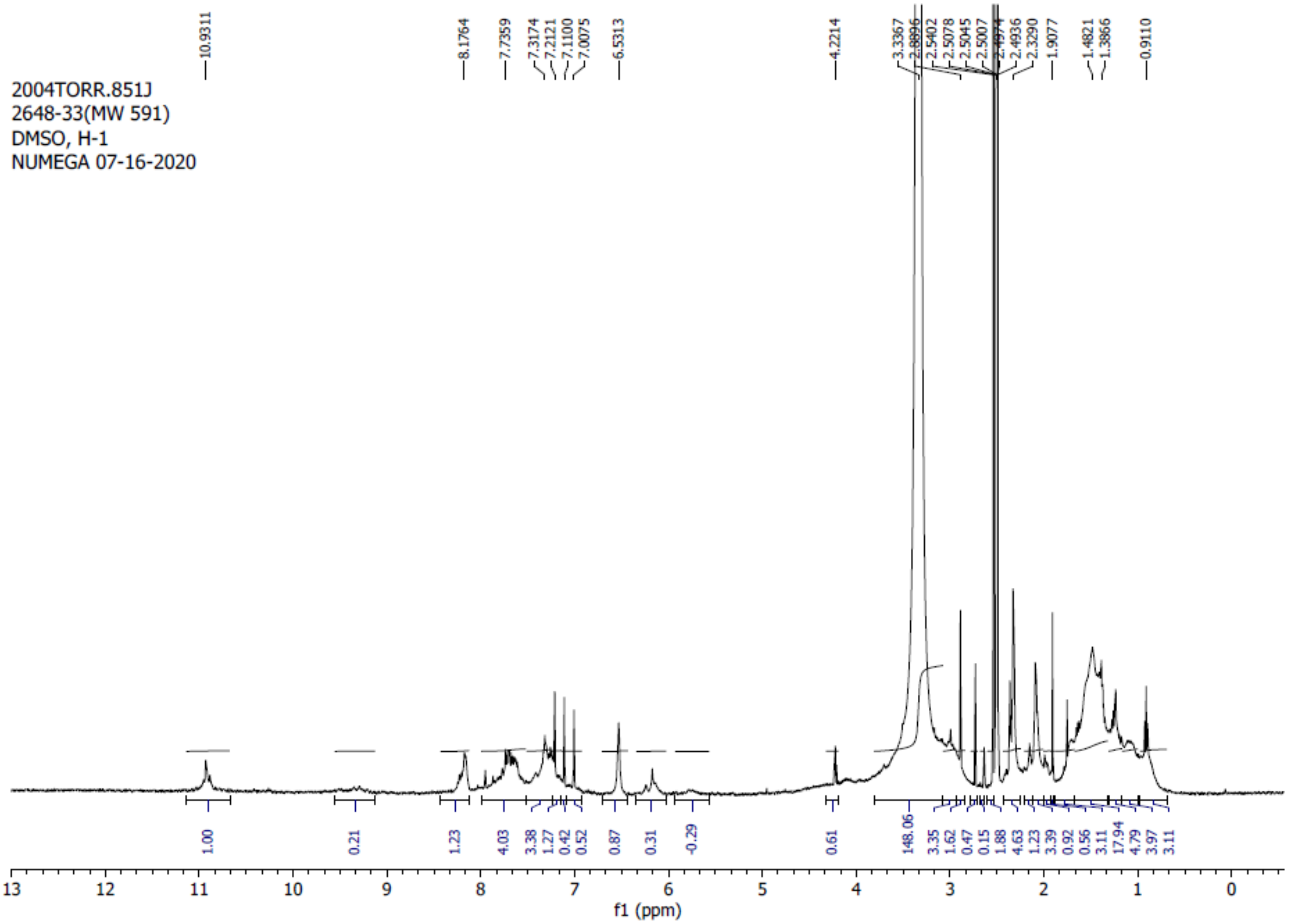
2004TORR.851J
2648-33(MW 591)
DMSO, H-1
NUMEGA 07-16-2020
10.9311
8.1764
7.7359
7.3174
7.2121
7.1100
7.0075
6.5313
4.2214
3.3367
2.8896
2.5402
2.5078
2.5045
2.5007
2.4974
2.4936
2.3290
1.9077
1.4821
1.3866
0.9110
1.00
0.21
1.23
4.03
3.38
1.27
0.42
0.52
0.87
0.31
-0.29
0.61
148.06
3.35
1.62
0.47
0.15
1.88
4.63
1.23
3.39
0.92
0.56
3.11
17.94
4.79
3.97
3.11
13 12 11 10 9 8 7 6 5 4 3 2 1 0
f1 (ppm)

Supplementary Figure S4. Cytotoxic effect of compounds 2648-33 and 2648-40 on human erythrocytes. Compounds tested at shown concentrations (Conc.). Positive control is 0.1 % Triton X-100. Negative controls are PBS and 0.25% DMF. Bars are averages of duplicates and error bars are Standard Deviations.

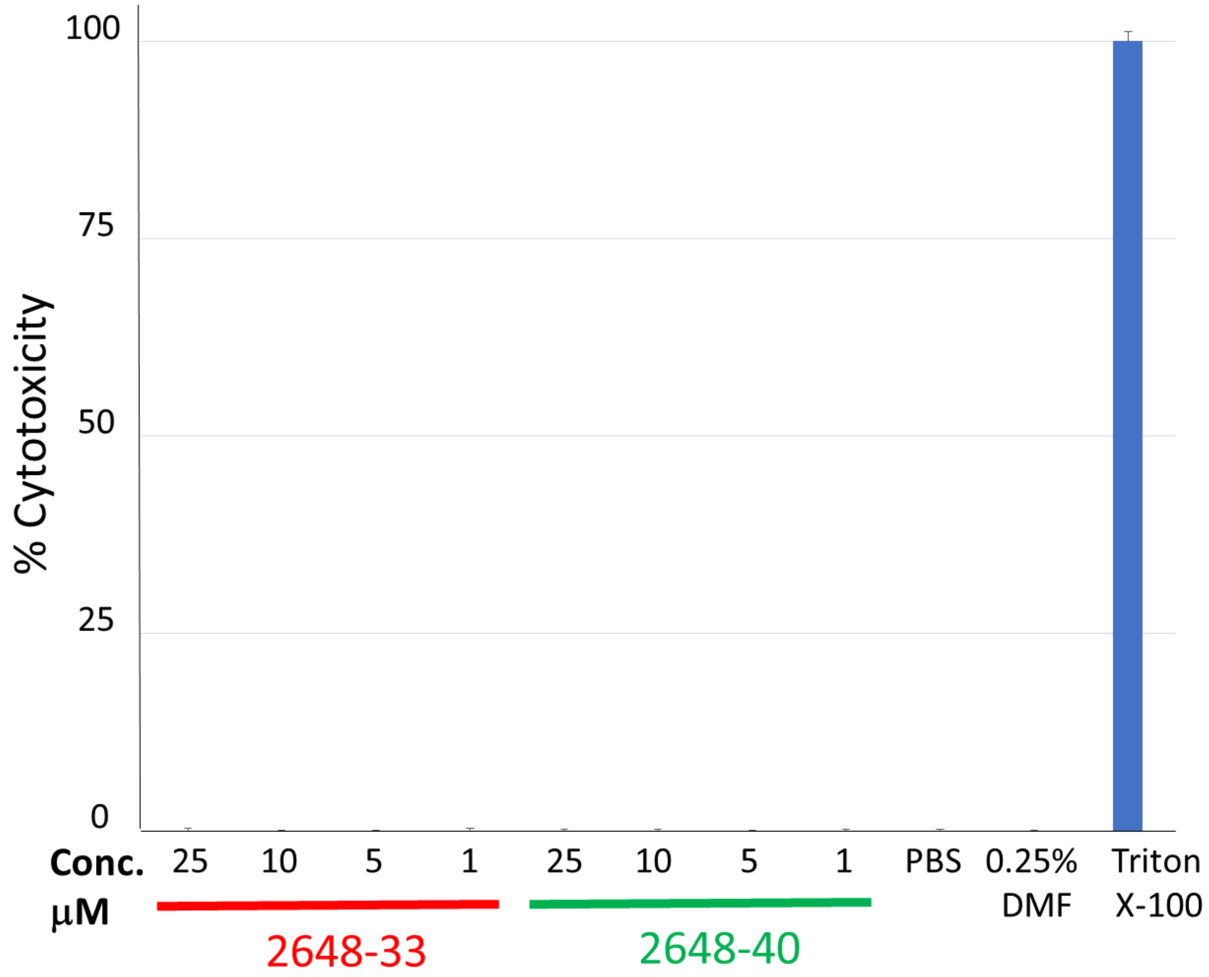

Supplement: Supplementary file 1 [file ijms-22-05659-s001.zip › ijms-1221657-supplementary.pdf]
